# Medical Society of the County of Erie

**Published:** 1898-08

**Authors:** F. C. Gram


### MEDICAL SOCIETY OF THE COUNTY OF ERIE.

Reported by F. C. GRAM, M. D., Secretary.

*Special Meeting, held June 23, 1898.*

A SPECIAL meeting of the Medical Society of the County of Erie was called by the officers to consider the existing municipal ordinance relating to infant feeding bottles. The Vice-president Dr. J. B. Coakley called the meeting to order at 8.45 p. m., Thursday, June 23, 1898, and the secretary read the call for the meeting.

The chairman asked the further pleasure of the meeting, whereupon the following resolutions were offered by Dr. R. H. Hopkins, seconded by Dr. Snow :

WHEREAS, The Medical Society of the County of Erie in common with the profession of medicine and other well-informed and right-minded persons maintain the following principles ; to-wit :

That the preservation of the public health is the first duty of the state.

That the prevention of the communicable diseases is the plain and imperative duty of the sanitarian.

That the responsibility of the state to afford protection to its citizens increases with the ignorance, carelessness and helplessness of such citizens.

That of all our objects of protection, none are more helpless, or in more constant need of wise and discriminating care than the newly born who must be reared by artificial feeding.

That the death-rate among the artificially fed newly born marks the level of the sanatory enlightenment of the community and

WHEREAS, Competent medical opinion has, and does, unhesitatingly and unanimously condemn a certain type of feeding bottles, in frequent use, and

WHEREAS, Inspired by such well-founded conviction, ordinances forbidding the sale of this apparatus were recently enacted by the proper authorities of this city ; and

WHEREAS, The relations between the professions of medicine and pharmacy are, and should be most reciprocal and harmonious, the pharmacist being a conspicuous citizen, learned in medicine, with singular opportunities to form and develop a right public opinion, therefore, be it

*Resolved*, That we commend to the druggists and pharmacists of our city our health ordinances in general, and with greater particularity the ordinance forbidding the sale of a certain class of feeding bottles as timely, wise, and in the best interests of the public health, and that we invite them to join us in supporting these ordinances with their earnest and constant efforts.

*Resolved*, That we commend to all those in authority the subject of the preservation of the lives of the newly born, and those of tender years as of the highest practical importance, as deserving this incessant and sleepless vigilance.

*Resolved*, That we approve in the most hearty and unqualified terms of the ordinance prohibiting the sale of any nursing bottle that has connected therewith a rubber tube, hose, or similar contrivance.

Dr. H. R. HOPKINS, in support of the resolutions, said that the interest of this society in sanitary measures had long been well established. Our knowledge of sanitary matters is greater than that of a majority of our inhabitants, and as our knowledge is greater so our responsibility increases. He called attention to the great infant mortality and held that this society should do all within its power to uphold the hands of the health authorities in enacting and enforcing ordinances beneficial to health.

Dr. IRVING SNOW said that the long-tubed nursing bottle had killed more than the modern rifles. When a child must be fed artificially it becomes necessary to exercise the greatest care. The long-tubed bottles are preferred by mothers because they are the most convenient. He objected to their use, first, because of the tendency to overfeed, which is not the case where the nipple only is used; secondly, because it continually poisons the milk, and thirdly, because it is impossible to keep the tubes clean. They are condemned by every author on sanitary measures for the same reasons.

Dr. W.W. Potter thought the resolutions were very considerately phrased. He believed the opposition of the dealers to the health ordinance was due to a misunderstanding. The pharmacists ought to be our coadjutors on so vital a question.

Dr. Lucien Howe requested the secretary to read the ordinance in question, being part of Section 81, Chapter XXV., as follows: "It shall be unlawful for any person or persons to use or to engage in the sale of any bottle, mechanism or other device for the artificial feeding or nursing of infants or children under three years of age, that has connected therewith a rubber tube, hose or similar contrivance."

President Howe then stated that owing to the recent discovery by the health authorities that long-tubed nursing bottles could still be purchased in many places, in violation of the ordinance, he had deemed it his duty to call a special meeting of this society, which had always been recognised as a guardian of public health, and here is something which is acknowledged by all authorities as detrimental to health and life.

Remarks in further support of the resolutions were made by Drs. Benedict, W. D. Green, Bayliss, Ring, Pohl and Heath, after which the preamble and resolutions were unanimously adopted.

The meeting then adjourned.

*Special Meeting, held July 11, 1898.*

IN MEMORY OF DR. JOHN BOARDMAN.

After the adjournment of the Academy of Medicine at 10 p. m., July 11, 1898, the members of the Medical Society of the County of Erie convened for a memorial meeting on the death of Dr. John Boardman, an ex-president of this society, who died July 9, 1898, aged 70 years.

President Howe paid a high tribute to the character of the deceased and stated that the meeting had been called to take appropriate action upon the death of one of our most distinguished members.

On motion the chair appointed a committee, consisting of Drs. Wyckoff, Thos. Lothrop, Putnam, Phelps and Dowd, to submit a memorial.

The committee reported the following :

WHEREAS : It has pleased Almighty God to terminate the suffering of our beloved colleague and friend, Dr. John Boardman, and

WHEREAS : By his christian fortitude and upright life, by his professional attainments, by his life-long unselfishness and his uniform courtesy to all with whom he came in contact, and by his devotion to duty he has left us an enduring example of what the physician should be ; and

WHEREAS ; His long years of patient suffering have won for him the sympathy and admiration of us all, therefore be it

*Resolved*, That the Medical Society of the County of Erie desires to place on record this testimonial of his worth as a man, a citizen and a physician.

*Resolved*, That we attend his funeral in a body.

C. C. WYCKOFF,
THOMAS LOTHROP,
W. C. PHELPS,
J. HENRY DOWD,
JAMES W. PUTNAM.

In speaking on the adoption of the memorial Dr. T. M. JOHNSON said that he had known Dr. Boardman since 1863, as an excellent student, a painstaking and hard-working practitioner and a model man.

Dr. F. W. ABBOTT had the privilege of becoming acquainted with the deceased while a student attending clinics at the Sisters' Hospital, where Dr. Boardman was then an attending physician. He knew him as a retiring, modest and self-depreciating practitioner, who always exercised the greatest care in his work.

Dr. SHERMAN said that the sentiments contained in the memorial were really mild expressions of the great fortitude of the deceased, and he briefly gave some incidents connected with his last illness.

Dr. GEO. W. YORK had the privilege of rendering alleviation to the suffering of the deceased during his illness. Although suffering intensely he rarely gave any indication of the fact or asked for any relief, bearing all with a determination that was remarkable. He spoke at length on the subject.

Dr. WYCKOFF knew Dr. Boardman since the latter was a student. He was by nature, instinct and practice, a thorough gentleman, a good diagnostician and a successful practitioner. His character would bear the closest scrutiny.

Dr. PUTNAM remembered Dr. Boardman for the kindly counsel and encouragement which he always gave to students and young practitioners. He also spoke of his christian character.

Dr. PHELPS said little could be added to the kind and loving tributes just paid to the deceased colleague's memory, whom he had known for upwards of thirty years, but nothing in the line of praise could even do him justice.

The memorial was adopted and adjournment followed.

---

